# Women’s experiences with unplanned pregnancy and abortion in Kenya: A qualitative study

**DOI:** 10.1371/journal.pone.0191412

**Published:** 2018-01-25

**Authors:** Ruvani T. Jayaweera, Felistah Mbithe Ngui, Kelli Stidham Hall, Caitlin Gerdts

**Affiliations:** 1 Ibis Reproductive Health, Oakland, California, United States of America; 2 Fortress of Hope Africa, Nairobi, Kenya; 3 Behavioral Sciences and Health Education, Rollins School of Public Health, Emory University, Atlanta, Georgia, United States of America; National Academy of Medical Sciences, NEPAL

## Abstract

**Background:**

Safe and legal abortions are rarely practiced in the public health sector in Kenya, and rates of maternal mortality and morbidity from unsafe abortion is high. Little is known about women’s experiences seeking and accessing abortion in informal settlements in Nairobi, Kenya.

**Methods:**

Seven focus group discussions were conducted with a total of 71 women and girls recruited from an informal settlement in Nairobi. The interview guide explored participants’ perceptions of unplanned pregnancy, abortion, and access to sexual and reproductive health information in their community. Thematic analysis of the focus group transcripts was conducted using MAX QDA Release 12.

**Results:**

Participants described a variety of factors that influence women’s experiences with abortion in their communities. According to participants, limited knowledge of sexual and reproductive health information and lack of access to contraception led to unplanned pregnancy among women in their community. Participants cited stigma and loss of opportunities that women with unplanned pregnancies face as the primary reasons why women seek abortions. Participants articulated stigma as the predominant barrier women in their communities face to safe abortion. Other barriers, which were often interrelated to stigma, included lack of education about safe methods of abortion, perceived illegality of abortion, as well as limited access to services, fear of mistreatment, and mistrust of health providers and facilities.

**Conclusions:**

Women in informal settlements in Nairobi, Kenya face substantial barriers to regulating their fertility and lack access to safe abortion. Policy makers and reproductive health advocates should support programs that employ harm reduction strategies and increase women’s knowledge of and access to medication abortion outside the formal healthcare system.

## Introduction

Unsafe abortion continues to be a persistent public health problem, and is responsible for 13% of all maternal deaths globally [1]. The World Health Organization reported that 21.6 million unsafe abortions occurred in 2008, with the majority of these occurring in developing regions [1]. In countries like Kenya, where abortion is restricted and rates of unintended pregnancy are high (40%) [2], women are often unable to access safe abortion [3]. As a result, women are forced to self-induce or seek abortions from clandestine providers in unsafe conditions, with consequences ranging from mild complications, to life-long morbidities, to death [4].

Eastern Africa has one of the highest rates of unsafe abortion among sub-regions worldwide, with unsafe abortion responsible for an estimated 18% of maternal deaths [1]. In Kenya, incidence of induced abortion and maternal mortality from unsafe abortion is estimated to be much higher: a recent study in Kenya on induced abortion estimated an annual abortion incidence of 48 abortions per 1,000 women of reproductive age [5], compared to an estimated annual abortion incidence of 34 per 1,000 in the East Africa region [6]. Several other studies have reported that at least one third of maternal mortality in Kenya is due to unsafe abortion [7, 8]. As underreporting is common, it is possible the true abortion rate and contribution of unsafe abortion to mortality are much higher [2].

In 2010, Kenya revised its constitution to permit abortion if “in the opinion of a trained health professional, there is need for emergency treatment, or the life or health of the [pregnant woman] is in danger, or if permitted by any other written law” [2]. The expansion of the law to permit abortions under a broader “health” exception was intended to expand access to safe and legal abortion in the public setting [9]; however, lack of clinical guidelines has led to ambiguity over the law’s interpretation, and qualified providers remain untrained in the provision of safe abortion [2, 10]. Thus, safe and legal abortions are rarely practiced in the public setting, as both women and providers fear legal repercussions [4, 11].

Further complicating matters, abortion is highly stigmatized in Kenya [4, 11–14]. Previous qualitative research in Kenya demonstrates that women who are known or thought to have procured abortions face considerable social stigma [4, 11–14]. Participants in these studies often link this stigma to why women choose to seek abortions in secret, often utilizing unsafe methods including but not limited to: insertion of objects such as crochet needles or catheters into the uterus, deliberate bodily injury, and ingestion of herbs, medications, cleaners, and juices [4, 11, 13, 15]. In one study of women seeking post abortion care at public hospitals in Kenya, participants cited fear of stigma and potential loss of privacy as primary reasons why women preferred to self-induce or seek abortions from unqualified providers recommended through their social networks rather than from hospitals [15]. Given women’s reliance on these life-threatening methods, interventions that seek to expand women’s knowledge of and access to safe abortion methods are sorely needed, while simultaneously taking into account their concerns around exposure to stigma and mistrust of the public health system.

Studies have shown that in restrictive environments, women who are able to access medication abortion (misoprostol and/or mifepristone) and post-abortion care services can be empowered to manage their own abortions with low rates of complications [16, 17]. Misoprostol is widely available in many countries with restrictive abortion laws as treatment for gastric ulcers; and in many different contexts women’s advocacy and health organizations have undertaken the task of making information available on how to safely administer misoprostol, according to World Health Organization protocols, for pregnancy termination [16, 18–20]. One of the most well-known models of this kind of abortion harm reduction is the model that originated in Uruguay, where, for decades, clinicians have provided women who were seeking abortions with information on how to use misoprostol correctly and safely [16]. However, in communities where women do not have the option to seek care from the formal health system, additional research is needed to better understand ways to increase access to safe abortion in such settings.

While previous research has documented some of the barriers that women face to safe abortion in central and western Kenya [11, 13], little is known about women’s experiences seeking and accessing abortion in informal settlements in Nairobi, where nearly 1 million women of reproductive age reside [21]. Understanding the ways in which women in informal settlements in Nairobi access sources of information on sexual and reproductive health, and their articulated information needs, could contribute to the creation of harm reduction strategies that meet the needs of the community and increase women’s access to safe abortion.

To address this gap, we conducted a secondary analysis of qualitative data collected by a grassroots organization serving informal settlement communities in Nairobi to explore women’s experiences and perceptions regarding abortion in their community, barriers women face in accessing safe abortion, and women’s perceptions of their own information needs and those of women in their community around sexual and reproductive health.

## Methods

### Sample and design

Data for this secondary analysis come from ongoing monitoring and evaluation activities conducted by a grassroots organization that seeks to empower marginalized girls in Nairobi, Kenya. Data were originally collected as part of an internal evaluation of the women’s empowerment and sexual and reproductive health and rights education initiatives delivered by the organization. Focus group participants were women and girls of reproductive age living in an informal settlement in Nairobi, Kenya. The name of the organization as well as the name of the informal settlement are withheld to protect confidentiality. The informal settlement has a population of around 100,000, and is characterized by sub-standard housing, limited access to electricity and water sources, as well as cramped living conditions. The organization primarily conducts activities in communities with limited access to safe abortion or other sexual and reproductive health services; participants in these activities are typically women and girls of reproductive age and are invited by community organizers or through word of mouth by peer recruiters.

In August 2015, the organization conducted seven focus group discussions comprised of 9–13 women each and a total of 71 women and girls aged 15 to 35. Participants were recruited from current or previous participants in the organization’s empowerment and sexual and reproductive health programming initiatives, and were a convenience sample of women and girls who elected to attend a series of workshops held by the grassroots organization in August 2015. Participants provided verbal consent. Focus groups were held in a secure, private location to ensure confidentiality and privacy and where participants could speak without danger of being overheard. The organization does not routinely collect detailed demographic data on participants in their programs; as these focus groups were part of standard programming and evaluation conducted by the organization, the only demographic information collected was age and marital status. No names or identifying information were recorded. Participants were each given between 100–500 Kenyan shillings ($1–$5 US Dollars) at the conclusion of the focus group discussion, depending on how far they had to travel.

Trained staff members from the organization facilitated the focus groups. Facilitators used a semi-structured open-ended discussion guide; key informant interviews with staff members and providers who serve women in this community, as well as a review of the literature informed the development of the study instrument. The interview guide was designed in order to elicit responses around women’s experiences in relation to the organization’s key programmatic areas: experiences with unplanned pregnancy, experiences with abortion, and women’s need for information on sexual and reproductive health in their community. Participants were probed further for their perceptions of a) the barriers that women in their community face to obtaining safe abortions, b) how women who obtain abortions are treated, and c) women’s current and preferred sources of information on sexual and reproductive health topics. As these topics are potentially stigmatizing, women were asked to report on the general experience of women in their community rather than describe their personal experiences. Organization staff members not involved in the development of the discussion guides pre-tested the instrument in order to ensure clarity and comprehension.

All participants consented to being audio-recorded. Focus group discussions, ranging from 40 to 80 minutes, were conducted in Swahili, digitally recorded, transcribed verbatim, and translated into English by an external transcriber. The data were provided to authors as anonymous records. As the authors only had access to data that was completely de-identified, the study did not need ethics approval; this determination was made by the Principal Investigator (CG) following the guidelines set forth by the University of California, San Francisco Institutional Review Board. Details about the development of the interview guide, recruitment, and training of data collectors were provided to the authors by the grassroots organization.

### Analysis

Thematic analysis of the focus group transcripts was conducted using MAX QDA Release 12 [22]. One author reviewed the focus group transcripts using a phenomenological approach to develop codes and code definitions. After reviewing three focus groups, identified codes were consolidated and summarized in a codebook. These codes were then applied to relevant blocks of text in the remaining transcripts. If new codes emerged in the data, the codes were added to the codebook, and all transcripts were reviewed again in an iterative process. Codes were summarized in analytical memos, and illustrated using direct quotes from the participants. All authors reviewed the codebook and memos, and discussed emergent themes. Conversations about the findings with Kenyan partners allowed for discussion of alternative explanations, better understanding of the local context, and opportunities to address issues of reflexivity. Key themes were identified, with the main findings discussed below.

## Results

### Characteristics of participants

The mean age of participants was 20.7 (std dev 4.0, range 15–35 years). The participants were primarily students, unemployed, or working as casual labors in city industries. Of the 56 participants with completed marital status data, 28.6% were married. No other demographic information was collected from study participants.

### Factors influencing women’s abortion experiences

Participants described a variety of factors that influence women’s experiences with abortion in their communities. According to participants, limited knowledge of sexual and reproductive health information and lack of access to contraception led to unplanned pregnancy among women in their community; participants cited stigma and loss of opportunities that women with unplanned pregnancies face as the primary reasons why women seek abortions.

#### Limited knowledge of sexual and reproductive health information leads to unplanned pregnancies

Participants in all seven focus groups identified and expressed the need for information and education on sexual and reproductive health and rights in their communities as a means of preventing unplanned pregnancy, especially for socially disadvantaged young women: *“(Unplanned pregnancy) is something that happens almost daily because*, *one*, *people are not educated*. *So*, *because they are not educated*, *and again parents do not just talk to their children*, *it’s normal for children in the ghetto to get pregnant*.*”* (Participant from FGD 3). Other consequences of a lack of sexual and reproductive health and rights education included early marriage, with one participant linking her lack of information to loss of future opportunities: “*You find someone like me got married very early because I had no information*. *Maybe I could be very far today*.*”* (Participant from FGD 2). When prompted by facilitators to list what kinds of information they thought would be most valuable and useful to them and women in their community, highly cited topics were information on contraception, gender-based violence, prevention of sexually transmitted infections and HIV, and sexuality.

#### Limited access to and use of contraception leads to unplanned pregnancies and stigma

Participants cited contraceptive failure as one reason why women in their community have unplanned pregnancies. Although participants were aware of methods to prevent pregnancy and their availability at public health facilities free of charge, fear of stigma and discrimination from health providers, poor quality of services, and lack of availability or access to wider family planning options were described as primary drivers of women’s limited access to and use of contraception. For example, health facilities frequently experience contraceptive stock-outs, causing gaps in their ability to provide methods and services: “*Some of them* [methods] *are free in city council health facilities*. *But if you go there late*, *like in the middle of the day*, *and they tell you come tomorrow*, *that was the day the injection was due*.” (Participant from FGD 2).

As a result of these barriers, unplanned pregnancy was a relatively frequent occurrence in this community: *“Many times we girls get pregnant without a plan*, *you just find yourself pregnant*.*”* (Participant from FGD 2). Unplanned pregnancy-associated stigma was commonly described, particularly among young unmarried women, who were socially isolated from their friends and family and gossiped about: “*They put you in an isolation…if the victim* [woman with the unplanned pregnancy] *is your friend*, *your mother tells you not to walk around with her*.” (Participant from FGD 3). Some women experience denial and refusal from their partners: “*Your boyfriend makes fun of you*, *in fact*, *he denies it saying*, *‘It’s not mine*.*’*” (Participant from FGD 1). Participants also described emotional stress and fear as consequences of unplanned pregnancy: “*You get stressed out and left with no other option*, *you commit suicide telling yourself you’re letting go*.*”* (Participant from FGD 4). Participants described a myriad of negative consequences of unplanned pregnancies that women in their communities face, such as early marriage and loss of employment and educational opportunities: *“Others*, *if they are at school when they get to this point*, *their education comes to a standstill and they do not continue*.*”* (Participant from FGD 6). Another participant summarized the diminished life goals women with unplanned pregnancies face: “*Their dreams are shattered*.*”* (Participant from FGD 3).

#### Unplanned pregnancies lead to abortion and further stigma

Participants linked the negative consequences of unplanned pregnancy to why many women seek abortions. However, abortion was viewed very negatively by communities, and, as such, participants reported that women are highly judged and stigmatized if they are known or suspected to have procured abortions. Stigma manifested most commonly as gossip, verbal and physical abuse, being socially ridiculed or “laughed at,” and accusations of engaging in sex work or prostitution. Stigma also led to perceptions that abortions were the results of infidelity or of being unfaithful to their partners, and in conflict with the gendered social expectations of motherhood. One participant described this: “*You will be breaking your own marriage because there is no man who doesn’t want a child; you were married to give birth*.” (FGD 5). Other forms of stigma included social isolation, accusations of murder, and lack of respect. “*Some are stigmatized and not allowed to say anything in the society*, *they are considered social misfits since they aborted*.*”* (Participant from FGD 7). Abortion stigma brought disgrace and shame to these women, their families and communities.

Secret-keeping and non-disclosure was a result of women attempting to avoid abortion stigma: “*Some* [gossip] *are caused by our friends*. *You tell them you are pregnant and they spread the news*.*”* (Participant from FGD 3). Many participants echoed this sentiment: “*Let me tell you*, *if I got pregnant and had an abortion*, *it would be my secret*.” (FGD 1). Participants described how people in the community believed that women who have abortions could be identified by clues, such as physical or behavior changes. However, perceptions were not always correct and clues could be misleading, as described by one participant: “*I have a personal experience when I was in Form Three*, *my breasts appeared quite early and people used to say this girl has big breasts she has procured an abortion*, *and I was still a virgin and I had not had sex*.” (FGD 2).

### Barriers to safe abortion

Participants articulated stigma as the predominant barrier women in their communities face to safe abortion. Other barriers, which were often interrelated to stigma, included lack of education about safe methods of abortion, perceived illegality of abortion, and limited access to services, fear of mistreatment and mistrust of health providers and facilities.

#### Stigma leads women to use unsafe methods to end pregnancies

Secret-keeping, non-disclosure, and lack of awareness of safe methods were described as primary reasons why women choose to forgo abortion in the health services sector and rather undergo riskier procedures or self induce. Participants generally described women as knowing the significant dangers that unsafe abortion carries, including risk of method failure and ongoing pregnancy, delays in the treatment of complications, and often death. One participant described this risk: “*So*, *if I find the Jik* [liquid bleach] *and Omo* [laundry detergent], *I use it alone because I don’t want my mother to find out*. *Someone comes* [to help] *at the very last minute when you realize you are dying*. *Some* [women] *say something at the last minute*, *some even die before they tell anyone*.” (FGD 1). Another described why, even despite the risks, that women are forced to resort to unsafe methods: *“Why*? *Because we’ve been saying here how we use hangers and crotchets*. *We wouldn’t be using them if there were safe methods*.*”* (Participant from FGD 3). Indeed, risks were considered calculated as women are desperate to end their pregnancy: *“In that situation you have decided either to die or not*.*”* (Participant from FGD 3).

Participants cited a variety of methods that women in their communities used to terminate pregnancies, all of which fall under the World Health Organization definition of *unsafe abortion*[23] (Table 1). The two most commonly cited methods were the ingestion of concentrated tea leaves, undiluted juices, or large amounts of soda. One participant said, “*I know of a friend who took some Quencher* [fruit flavored drink] *that was not diluted and then she bled excessively; by the time she was getting to the hospital*, *she was dead*.” (FGD 2). Others cited solvents and cleaning solutions such as bleach and laundry detergent and traditional herbal medicines as commonly used ingestible methods. Collectively, these different methods were discussed in all focus groups. Participants reported that these methods were relatively inexpensive (less than $0.50 USD), widely available, and easy to obtain in small quantities. However, these ingestion methods were regarded as unsafe: *“If someone uses Jik* [liquid bleach] *it can kill*. *It almost works like the way it bleaches clothes—it can also bleach your uterus—it can even cause tears on clothes*, *it means it is dangerous*.*”* (Participant from FGD 2).

**Table 1:** Methods reported by participants to self-induce abortions.

Insertion of…	Ingestion of…
Crochets Knitting Needles Straws Rubber Catheters Bottles	Concentrated Tea Leaves Boiled Roots Goat Feces Traditional Herbs Liquid Bleach Laundry Detergent Antiseptic Shoe Polish Undiluted Acidic Juice Soda Painkillers Deworming Drugs Unknown Medications

Some participants described insertion methods such as crochet needles, straws, coat hangers or other metal objects: “*Crotchets*, *they use them to hook the fetus*.*”* (Participant from FGD 3). Other insertion methods were mentioned: *“You can also insert a pipe/straw at the uterus and prick it*, *it will come out as long as air get inside the uterus*.*”* (Participant from FGD 6). While many of these methods were possible to procure and use alone, participants also reported that women went to chemists (local pharmacists) or traditional birth attendants/midwives to procure such methods, as one participant said, “*For example*, *there is a woman who helps women with childbirth in the village and does unsafe abortions*, *she uses straws*, *bottles*.” (Participant from FGD 3). Insertion methods were identified as particularly unsafe due to trauma to the uterus: *“Some are treated badly by local traditional birth attendants*, *they insert crude things and the womb comes out*. *It can damage your uterus and you won’t be pregnant for the rest of the your life*.*”* (Participant from FGD 2).

Only one participant in all seven focus groups spontaneously mentioned Medabon (mifepristone and misoprostol) as a method of abortion. When prompted by FGD facilitators, medication was discussed in only three groups. Participants in those groups referred generally to pills that one could obtain from a pharmacist, chemist or traditional birth attendant, though some reported an uncertainty of what type of medication women were given: “*You just go and buy tablets for abortion and sometimes you don’t know which ones to use and how to use them*.” (Participant from FGD 2). Participants in two focus groups mentioned a distrust of these sources and methods, with the common perception that ineffective or counterfeit medications are one reason for abortion failure: *“One tells you that if you take these four family planning pills*, *you’ll get rid of the pregnancy*. *You try*, *it fails*.*”* (Participant from FGD 1). Participants also reported off-brand use of known medications such as Panadol (acetaminophen) and deworming medication to induce abortion: “*It aborts because it is usually written*, *‘do not use if pregnant*.’” (Participant from FGD 3).

Overall, participants viewed all of the aforementioned methods, including abortion medications, as unsafe, unreliable, and carrying severe adverse consequences for women in their community. In every focus group, women unanimously agreed that abortion is unsafe and can lead to death. In all focus groups, women had examples of friends, neighbors, or other women in their community who died from abortion: “*Up to now three of them have already left us after carrying out abortion*. *We already buried them at Langata* [a cemetery].*”* (Participant from FGD 1). Other acute and chronic complications after inducing abortion that were specifically described by participants included bleeding, infertility, and repeat miscarriages.

#### Women lack information about safe abortion methods

When prompted by facilitators to list what kinds of information they thought would be most valuable and useful to them and women in their community, information on access to safe abortion was one of the most highly cited topics. In addition, participants discussed the importance of knowing more about their rights when accessing health care, including abortion services: “*They* [women] *need to know that they should not be abused by doctors when they go to hospital*.*”* (Participant from FGD 3). Regarding perceived appropriate sources of sexual and reproductive health information, participants mentioned the roles of community organizations, doctors and other healthcare providers, and schools in providing formal and informal education. In particular, participants expressed a desire for additional educational sessions from the grassroots organization: *“We need more of this kind of education that you are giving us like today*, *keep doing this*.*”* (Participant from FGD 7).

When asked by facilitators whether women would feel comfortable talking to a counselor over the phone about access to safe abortion and other sexual and reproductive health concerns, participants reported that if women were aware of a hotline, they would call it: *“In* [this neighborhood] *many girls are in trouble*, *and now when they know there is this hotline they will call*, *because there are some who are used badly by their husbands*.*”* (Participant from FGD 2). Participants also reported being comfortable with asking someone they don’t know directly for advice because of the stigmatization of abortion and unplanned pregnancy in their community: *“I’d rather* [talk to] *one you don’t know because a friend might go and spread the news out there or they don’t even believe all your words*.*”* (Participant from FGD 5). However, lack of money was a major concern cited by participants, even for potentially low-cost interventions such as a sexual and reproductive health phone hotline: *“But then they will find out it is not free and they won’t call again*.*”* (Participant from FGD 5).

Participants also described peers and family as informal sources for abortion information, but were aware that some information received from such sources can be inaccurate or unsafe: “*You will talk to a friend*, *and they advise you to use a crotchet because you don’t have information*.*”* (Participant from FGD 3). Overall, participants expressed women’s eagerness for education and training on sexual and reproductive health topics for themselves and so they can disseminate accurate information to their friends and community members. Indeed, participants discussed the support that women can and should provide for one another. Participants in FGD 1 described this role of social networks, *“You know in the ghetto there are problems and wherever I find help I would obviously want my friend to find the same*.*"* (Participant 1). *“Like in my case*, *my friends are gone*, *I’m only left with* [name of friend withheld], *there’s no way I’d hear of good information and fail to let her know*.*”* (Participant 2).

#### Women perceive abortion to be illegal

The legal status of abortion was not known or understood among women in participants’ communities; the perceived illegality was a primary reason why women chose not to attend health facilities for abortion services. As one participant described, “*Maybe what you are going to do there might bring you problems*, *sometimes you go and sit with the doctor thinking that he is going to assist you*, *you tell him everything and at the end of the day you are arrested*.” (Participant from FGD 5). Participants further described the perception that anyone who helped women obtain abortions would be imprisoned.

#### Women have limited access to abortion services

Many participants described women’s financial barriers to abortion services in the healthcare sector. Some believed that hospitals offer clandestine services that are available to a woman only if she is able to bribe a doctor: “*In fact I know of one very big one and he is a doctor at* [Hospital A], [he’s] *so corrupt*, *he helps people abort when you bribe him*, *you see*.” (Participant from FGD 3). The extremely high cost for abortions at hospitals was mentioned repeatedly as a barrier to seeking care from facilities: “*So*, *right now I can go to* [Hospital A] *and abort*, *yes*. *As long as you have money*. *They tell you to pay twenty thousand shillings* [~$200 USD] *and you pay it*.*”* (Participant from FGD 5). Participants cited cost as a reason for why women engage in unsafe methods: “*The costs at* [Hospital A] *and* [Hospital B] *is high*, *that is why we take Omo* [laundry detergent].” (Participant from FGD 6). Notably, the costs of abortion from health facilities was significantly higher than the cited costs of obtaining an abortion from midwives or chemists, which ranged from 1,500 to 3,000 Kenyan shillings ($15 to $30 USD). However, there was some disagreement over whether chemists and midwives were qualified abortion providers. Some participants reported that chemists are licensed doctors, while other women acknowledged, *“They are just quacks here*.*”* (Participant from FGD 1).

Participants also generally believed that abortions were only available at hospitals for women with life-threatening conditions: *“I remember when I was about to deliver*, *we were taught that some pregnancies can be fatal*, *you see you can be tested*, *some can be risky to your health*, *in that case an abortion can be carried out in a hospital*, *you see*. *There are some that are allowed and some that are not*.” (Participant from FGD 5).

Additionally, geographic proximity to care was considered a barrier to accessing formal abortion services. One participant described the challenges of not having local services available: *“Us from* [this informal settlement] *have no hospital to take our problems to unless you have money to go to a private hospital*.” (Participant from FGD 3). Rather than travel long distances, manage logistical scheduling, and financial issues related to travel, participants summarized how, *“The majority of us use what is closest to us*.*”* (Participant from FGD 2).

## Discussion

One of the key aims of this analysis was to assess women’s experiences and perceptions of abortion in their community and their articulated needs regarding access to sexual and reproductive health information and services, in order to inform the larger evaluation’s goal of identifying potential community based interventions and broader policy change.

Previous research from Kenya suggests that women and girls face numerous barriers to controlling their fertility, experience high rates of unintended pregnancy [24–26], commonly encounter stigma associated with unintended pregnancy and abortion [4, 11–14], and resort to unsafe methods to end unwanted pregnancies [4, 11, 13, 15]. Findings from this study corroborate results from the existing literature, in addition to furthering our understanding of stigma as both a reason why women seek abortions and as a barrier to obtaining safe abortion services.

Despite the relatively frequent occurrence of unplanned pregnancy noted by participants in the study, unmarried women who experience a pregnancy face considerable social stigma. An estimated 13,000 Kenyan girls are forced to leave school each year because of unintended pregnancies [25]. Participants confirmed these findings by reporting that unmarried women with unplanned pregnancies are especially restricted with respect to their life choices, particularly in lack of schooling, access to life-changing opportunities, and diminished job prospects. Expanding adolescent’s access to contraception and safe abortion options may greatly enhance women’s opportunities for education, prosperity, and employment. Currently, however, abortion is viewed as women’s only recourse to avoiding the negative social and economic consequences of unplanned pregnancy.

The stigma of unplanned pregnancy is further compounded by abortion stigma: participants reported that fear of stigmatization drives women to procure abortions in secret. Participants highlighted secret keeping and non-disclosure as potentially dangerous given the lack of safety of methods used by women to end pregnancies. Previous literature has demonstrated the interplay between stigma and non-disclosure in a variety of contexts and legal settings;[27] findings from this study further demonstrate the universality of abortion stigma across a variety of different contexts. The role of communities in increasing and reinforcing abortion stigma is clear. Working with communities to develop interventions that seek to reduce abortion stigma and increase empathy in everyday community interactions for women seeking abortions, such as community-based distribution models, stigma reduction workshops, and awareness activities, may reduce women’s desire to procure abortions in secret, and reduce barriers to safe abortion.

The ambiguity behind Kenya’s constitution regarding abortion is another significant barrier to safe abortion; participants’ perception of all abortion as illegal not only prevents women from seeking care at health facilities, but also contributes to abortion stigma. As a result, women go to great lengths to keep their abortions secret, increasing the likelihood of using unsafe methods such as ingesting chemicals or inserting sharp objects into their uterus. Almost all participants believed that there were no safe methods of abortion; and most participants shared stories of friends, family members, and neighbors who died from abortion in their community. Women do appear to be aware of the dangers of using unsafe methods; and thus, education focused only on the dangers of unsafe abortion is unlikely to be a successful at reducing abortion related mortality and morbidity. A more holistic approach, including interventions aimed at increasing knowledge of safe abortion medications, increasing access to safe abortion, reducing unintended pregnancy, and addressing community-level abortion stigma is warranted.

It is likely that fear of mistreatment from formal providers and high costs lead women and girls to seek abortions from unqualified local providers or to resort to unsafe methods to terminate their own pregnancies. It is also likely that women’s fears about public healthcare facilities are not unfounded; therefore, interventions that encourage women to seek legal abortions through the formal health care system may also be ineffective unless coupled with Ministry of Health-led interventions targeted at improving provider knowledge and understanding of the law, and values clarification around abortion.

### Implications for research and policy

This study builds upon previous qualitative research on abortion in Kenya, and contributes further insight into women’s experiences with unwanted pregnancy and abortion stigma, their articulated needs and desire for sexual and reproductive health information, and the need for multi-pronged, community-based interventions. Interventions should build upon women’s cited desires for privacy and access to safe abortion within their communities, and should exploit women’s reliance on peer networks for information gathering. Results from this paper can be used to inform the development of harm reduction strategies, community empowerment initiatives to reduce mortality and morbidity from unsafe abortion, and policy change in Kenya.

In contexts like Kenya, where abortion stigma is pervasive, the legality is ambiguous, and trained abortion providers are in short supply, harm reduction strategies that seek to increase women’s access to misoprostol holds significant promise.[20] Possible harm reduction approaches include, but are not limited to: ensuring and expanding access to quality misoprostol, training pharmacists and other lay health workers on appropriate use and administration of misoprostol, supporting the development of hotlines and other mHealth interventions that provide women with confidential, accurate information on the use of medication abortion, and enhancing grassroots education and advocacy efforts to increase the awareness of and demand for medication abortion.[20] These interventions build upon women’s cited desires for privacy and access to safe abortion within their communities; interventions to highlight the availability and safety of medication abortion should leverage women’s reliance on peer networks for information gathering. Additional research in a variety of contexts is needed in order to assess the potential impact of these interventions on reducing maternal mortality from unsafe abortion and improving women’s experiences with abortion-seeking.

### Limitations

The goal of this research was to provide insight on women’s experiences with abortion in an informal settlement in Nairobi, Kenya; as such, results from this study may not be generalizable to a larger population. Furthermore, women and girls who participated in this study have had some interaction with the community-based organization, and thus may be more aware of sexual and reproductive health issues than non-participants from their community.

## Conclusions

Women in informal settlements in Nairobi, Kenya face substantial barriers to regulating their fertility and lack access to safe abortion. Given the high burden of maternal mortality from unsafe abortion in Kenya, policy makers and reproductive health advocates should support programs that employ harm reduction strategies and increase women’s knowledge of and access to medication abortion outside the formal healthcare system. Further research is needed to evaluate these strategies.

## Supporting information

S1 File IRB self-certification form. Self-Certification Form: Determining Whether Human Subjects Are Involved in Research When Obtaining Coded Private Information (Data) and/or Biological Specimens.
(PDF)
