# Supplementary material for: Women’s experiences with unplanned pregnancy and abortion in Kenya: A qualitative study
Source: PLoS One. 2018 Jan 25;13(1):e0191412. doi: 10.1371/journal.pone.0191412 (PMC5784933; doi:10.1371/journal.pone.0191412)
Supplement: S1 File — Self-Certification Form: Determining Whether Human Subjects Are Involved in Research When Obtaining Coded Private Information (Data) and/or Biological Specimens. (PDF) [file pone.0191412.s001.pdf]

University of California, San Francisco
Institutional Review Board

Self-Certification Form: Determining Whether Human Subjects Are Involved in Research When Obtaining Coded Private Information (Data) and/or Biological Specimens

Instructions:

1. Use this form if you need to provide funding agencies, administrators or collaborators with documentation that your research project does not require IRB review at UCSF. Keep a copy of the form in the PI's research file. Do **not** submit a copy to the IRB.

2. For help making this determination, review the Human Subjects Research Decision Tree and the Not Human Subjects Research guidance page. Contact the IRB at 415-476-1814 or IRB@ucsf.edu with questions.

3. Do not use this form for human stem cell research, which requires review by the GESCR Committee and may require IRB review.

Principal Investigator:

Name and Degree	Institution	Department
Caitlin Gerdts, M.H.S., Ph.D.	University of California, San Francisco	Advancing New Standards in Reproductive Health
**Mailing Address**	**Phone Number**	**E-mail Address**
1330 Broadway Suite 1100<br>Oakland, CA 94612	510 - 986 - 8967	caitlin.gerdts@ucsf.edu

Study/Grant Title/Award No.:

Aunty Jane Hotline Evaluation

If your research meets the following conditions, the use of de-identified or coded private information (data) and/or biological specimens does not meet the definition of a human subject and does not require IRB review at UCSF:

1. The research is not regulated by the Food and Drug Administration (FDA). **AND**

2. No one on the UCSF research team has access to identifiable information and one or more of the following apply (check all applicable boxes):

- [x] The researcher(s) receive de-identified data or specimens.
- [ ] The researcher(s) receive coded data or specimens AND one or more of the following apply:
  - [ ] The key to decipher the code is destroyed before the research begins.
  - [ ] The PI and holder of the key enter into an agreement prohibiting the release of the key under any circumstances. There are IRB-approved written policies for the repository or data management that prohibit the release of the key.
  - [ ] There are other legal requirements prohibiting the release of the key under any circumstances.

Principal Investigator's Certification:
I certify that the information provided in this application is complete and correct.

[signature]

Principal Investigators Signature

6/3/2015

Date
